# Osterix promotes the migration and angiogenesis of breast cancer by upregulation of S100A4 expression

**DOI:** 10.1111/jcmm.14012

**Published:** 2018-11-18

**Authors:** Shuang Qu, Jiahui Wu, Qianyi Bao, Bing Yao, Rui Duan, Xiang Chen, Lingyun Li, Hongyan Yuan, Yucui Jin, Changyan Ma

**Affiliations:** ^1^ Jiangsu Key Laboratory of Xenotransplantation Nanjing Medical University Nanjing China; ^2^ Department of Medical Genetics Nanjing Medical University Nanjing China; ^3^ Department of General Surgery The Affiliated Yixing Hospital of Jiangsu University Yixing China; ^4^ Department of Oncology and Lombardi Comprehensive Cancer Center Lombardi Comprehensive Cancer Center Washington District of Columbia

**Keywords:** angiogenesis, breast cancer, migration, osterix, S100A4

## Abstract

As a key transcription factor required for bone formation, osterix (OSX) has been reported to be overexpressed in various cancers, however, its roles in breast cancer progression remain poorly understood. In this study, we demonstrated that OSX was highly expressed in metastatic breast cancer cells. Moreover, it could upregulate the expression of S100 calcium binding protein A4 (S100A4) and potentiate breast cancer cell migration and tumor angiogenesis in vitro and in vivo. Importantly, inhibition of S100A4 impaired OSX‐induced cell migration and capillary‐like tube formation. Restored S100A4 expression rescued OSX‐short hairpin RNA‐suppressed cell migration and capillary‐like tube formation. Moreover, the expression levels of OSX and S100A4 correlated significantly in human breast tumors. Our study suggested that OSX acts as an oncogenic driver in cell migration and tumor angiogenesis, and may serve as a potential therapeutic target for human breast cancer treatment.

## 1 INTRODUCTION

Breast cancer is the most common malignancy and is a major cause of death among women worldwide.^1^, ^2^ Despite significant advances in early diagnosis, surgical intervention, and local and systemic adjuvant therapies, the mortality rate of breast cancer is still high, with distant metastasis being the main cause of death in patients with breast cancer.^3^ Spreading of tumor cells from the primary neoplasm to distant sites is a complex and multistep process, in which tumor cell migration and endothelial cell angiogenesis play important roles. However, the mechanism underlying this specific metastatic behavior remains poorly understood. Exploring the molecular mechanisms of cell migration and angiogenesis is essential to develop new diagnostic and therapeutic strategies for breast cancer.

Osterix (OSX), also known as SP7, is a zinc finger transcription factor that is usually expressed in osteoblasts to regulate osteoblast differentiation and maturation.^4^ The expression of OSX had been suggested to be limited to bone tissues; however, recent studies identified that OSX was overexpressed in various cancer tissues, such as osteosarcoma, prostate cancer and breast cancer.^5^, ^6^, ^7^ Nevertheless, there have been few studies on the role of OSX in carcinogenesis. OSX expression in osteoblasts is regulated by various factors, including bone morphogenetic protein 2 (BMP2), Msh homeobox 2 (MSX2), myogenic differentiation (MYOD), and distal‐less homeobox 5 (DLX5).^8^, ^9^, ^10^ However, its downstream signaling remains largely elusive. Although a recent study indicated that OSX could alter the expression profile of several metastasis‐associated genes, such as those encoding vascular endothelial growth factor (VEGF), matric metalloproteinase 9 (MMP‐9), β‐catenin, and E‐cadherin in human breast cancer cells,^7^ whether the expression of OSX is critical for cancer metastasis is unknown, and the underlying mechanism remains to be defined.

S100 calcium binding protein A4 (S100A4) is involved in a variety of physiological functions, such as cell motility, adhesion, proliferation, and metastasis.^11^, ^12^, ^13^, ^14^, ^15^ Mammary tumors from S100A4 transgenic mice displayed higher vessel density compared with non‐transgenic animals.^16^ Vessel density and S100A4 expression correlated positively in primary tumors from patients with breast cancer.^17^ S100A4 is a well established marker and mediator of metastatic disease.^18^, ^19^ However, the upstream molecular signaling pathway involved in S100A4‐mediated metastasis is less well defined.

In the present study, we investigated the role of OSX in the cell migration and tumor angiogenesis in breast cancer. We found OSX is highly expressed in metastatic breast cancer cells. Moreover, we identified that OSX could promote the breast cancer cell migration and tumor angiogenesis by increasing S100A4 expression, suggesting that OSX participates in breast cancer malignancy and may serve as a potential target for breast cancer therapy.

## 2 MATERIALS AND METHODS

### 2.1 Reagents and antibodies

Roswell Park Memorial Institute (RPMI)‐1640 medium, Dulbecco's modified Eagle's medium (DMEM), DMEM/F12, Opti‐MEM Reduced Serum Medium, and fetal bovine serum (FBS) were purchased from Gibco (Invitrogen, Carlsbad, CA, USA). General chemicals were purchased from Sigma (St. Louis, MO, USA) unless specifically mentioned. Short interfering RNA (siRNA) duplexes targeting the human *S100A4* gene and siRNA duplexes with nonspecific sequences were designed and synthesized by RiboBio (Guangzhou, China). Anti‐OSX antibodies were purchased from Abcam (Cambridge, MA, USA). Anti‐S100A4 and anti‐β‐catenin antibodies were obtained from Cell Signaling Technology (Beverly, MA, USA). Anti‐β‐actin, anti‐CD34, and horseradish peroxidase‐conjugated secondary antibodies were purchased from Santa Cruz Biotechnology (Santa Cruz, CA, USA). Anti‐CD44, anti‐VEGF, and anti‐CD31 antibodies were obtained from Proteintech (Chicago, IL, USA).

### 2.2 Cell culture

MCF 10A, MDA‐MB‐231, MCF7, T‐47D, MDA‐MB‐468, HUVEC and EA.hy926 cells were obtained from the American Type Cell Collection (Manassas, VA, USA). MDA‐MB‐231, T‐47D, and EA.hy926 cells were cultured in RPMI‐1640 medium; and MCF7, MDA‐MB‐468 and HUVEC cells were grown in DMEM. All culture media were supplemented with 10% FBS and 1% penicillin/streptomycin. MCF 10A cells were grown in DMEM/F12 medium supplemented with 5% horse serum, 20 ng/mL of epidermal growth factor (EGF), 0.5 mg/mL of hydrocortisone, 100 ng/mL of cholera toxin, 10 μg/mL of insulin, and 1% penicillin/streptomycin. All cells were incubated in a humidified atmosphere with 5% CO_2_ at 37°C.

### 2.3 Plasmid construction and RNA interference assays

A construct overexpressing human *OSX* was generated by ligating the full‐length open‐reading frame of *OSX* into the vector plenti‐EF1a‐GFP (GeneChem, Shanghai, China). The human *S100A4* cDNA was amplified by PCR and cloned into vector pRK5‐GFP (Genentech, South San Francisco, CA, USA). The primers used are listed in supplementary Table S1. To knockdown OSX expression, several independent short hairpin RNAs (shRNAs) against the human *OSX* gene were ligated into vector pGV248‐GFP (GeneChem), with a non‐targeting control sequence (shNC) serving as the control. Sequences of the shRNAs targeting *OSX* are shown in Table S2. The constructed plasmids were transiently transfected into breast cancer cells. Quantitative real‐time reverse transcription PCR (qRT‐PCR) was used to detect OSX expression and to validate the transfection efficiencies. The expression level of OSX was lowest in #1shRNA group. Therefore, #1shRNA was selected as the optimum shRNA for lentivirus packaging.

### 2.4 Stable transfections

High‐titer lentivirus was packaged in HEK 293T cells. The viral particles were collected by centrifugation at 48 hours post‐transfection, and applied to MDA‐MB‐231 cells in the presence of 5 μg/mL polybrene for 48 hours. Cells were selected using puromycin (3 μg/mL) for 2 weeks. Single colonies were screened by limiting dilution. Gene knockdown and overexpression were confirmed by Western blotting.

### 2.5 Mass spectrometry

Cells were solubilized with 7 mol/L urea, 2% thiourea, and 1% CHAPS (3‐[(3‐cholamidopropyl)dimethylammonio]‐1‐propanesulfonate). One milligram of protein was reduced with dithiothreitol, alkylated with iodoacetic acid, and digested with trypsin, as previously described.^20^ The tryptic peptides were desalted using a homemade C18 solid phase extraction column, dried in a Speed Vac (Eppendorf, Hamburg, Germany), and then resuspended in 100 μL of 100 mmol/L triethylammonium bicarbonate buffer. Procedures for dimethyl labeling were the same as those previously described.^20^ The labeled peptides were applied to an LTQ‐Orbitrap instrument (Thermo Fisher, Waltham, MA, USA) equipped with a Nano Aquity ultra‐performance liquid chromatography system (Waters, Milford, MA, USA) via a nanospray source for data acquisition. The tandem mass spectroscopy (MS/MS) spectra acquired from precursor ions were submitted to Mascot (version 2.3.01) using the following search parameters: The database searched was Uniprot proteome; the variable modifications were oxidation (M), Gln→Pyro‐Glu (N‐term Q); carbamidomethylation of cysteine was set at static modification; the enzyme was trypsin; the MS/MS tolerance was set at 10 ppm; and the false detection rate for peptides and proteins were all set to bellow 0.01.

### 2.6 Western blotting analysis

Cells were washed with phosphate‐buffered saline (PBS) and harvested in radioimmunoprecipitation assay buffer containing 10 mg/mL aprotinin, 5 mg/mL leupeptin, and 1 mmol/L phenylmethane sulfonyl fluoride. Protein concentrations were determined using the Pierce BCA protein assay kit. Equal amounts of protein were loaded onto a 10% SDS‐polyacrylamide gel for electrophoresis, and transferred to polyvinylidene difluoride membranes (Millipore, Billerica, MA, USA). The membranes were probed with the specific antibodies overnight at 4°C, and then incubated with secondary antibodies at room temperature for 1 hour. Immunoblots were developed using an enhanced chemiluminescene western blotting substrate kit (Pierce, Rockford, IL, USA) and exposure to Kodak BioMax MR film. Equal protein loading was confirmed by probing blots with antibodies against β‐actin.

### 2.7 RNA isolation and qRT‐PCR

Total RNA was isolated from cells using the Trizol reagent (Invitrogen), and cDNA was synthesized from 1 μg of total RNA using the Primescript RT Reagent (Takara, Otsu, Japan) following the manufacturer's instructions. The real‐time PCR was performed using FastStart Universal SYBR Green Master (Roche, Indianapolis, IN, USA) in a Roche LightCycler 96 Real‐Time PCR System. The amplification conditions were 95°C for 10 minutes, followed by 40 amplification cycles of 95°C for 10 seconds and 60°C for 30 seconds. Expression values were normalized to that of the *ACTB* gene (encoding β‐actin). The primers used for qRT‐PCR are listed in Table S3.

### 2.8 Cell migration assay

Cells (5 × 10^4^) were trypsinized, resuspended in serum‐free medium and then added into the upper chamber of the insert (Millipore). The total culture medium containing 10% FBS was added in the lower chamber. Cells were incubated in a humidified incubator at 37°C for 12 hours. Cells on the membrane were fixed with methanol and stained with crystal violet. The numbers of migrated cells were calculated from five randomly selected fields of views.

### 2.9 Preparation of conditioned medium

Cells were cultured overnight in a six‐well plate at a density of 4 × 10^5^ cells per well in a complete medium. The following day, cells were washed with PBS twice and incubated with Opti‐MEM Reduced Serum Medium for 24 hours. The supernatant medium was then harvested and centrifuged at 500 *g* for 10 minutes. Cell‐free conditioned medium (CM) was used for subsequent experiments.

### 2.10 Tubule formation assay

The microtubule formation assay was performed in 96‐well plates coated with 50 μL of matrigel (BD Biosciences, Bedford, MA, USA). HUVEC and EA.hy926 cells were seeded at 1 × 10^5^ cells per well and incubated with CM at 37°C for 24 hours. Five random selected fields of view were captured using a microscope. Tube lengths were assessed by drawing lines along the tube‐like structure and measuring the lengths of the lines in pixels using Image J software.

### 2.11 Angiogenesis assay on chicken chorioallantoic membranes

Fertilized white leghorn chicken eggs were incubated at 37°C under conditions of constant humidity. On embryonic day 3 (E3), 2‐3 mL of ovalbumin was gently aspirated and removed from the egg using a needle to create an air sac directly over the chicken chorioallantoic membranes (CAM), allowing its dissociation from the egg shell membrane. On E8, a small circular window was opened above the air sac, and then sealed with tape. Cell aliquots (20 μL) mixed with equal volume of high concentration matrigel were implanted onto the CAM. Images of CAMs were captured 7 days after implantation, and branching of the blood vessels were counted by two observers in a double‐blind manner. Assays for each treatment were carried out using 10 chicken embryos.

### 2.12 In vivo matrigel plug angiogenesis assay

Nude mice were manipulated and cared for according to NIH Animal Care and Use Committee guidelines in the Experiment Animal Center of the Nanjing Medical University (Nanjing, China). The protocol was approved by the Committee on the Ethics of Animal Experiments of the Nanjing Medical University. Female athymic BALB/c nu/nu mice, 3–4 weeks old (Model Animal Research Center of Nanjing University, Nanjing, China), were maintained under pathogen‐free conditions. Cell aliquots (100 μL) were mixed with an equal volume of high concentration matrigel, and then injected subcutaneously into the right flanks of the nude mice. The gel plugs were excised from nude mice 7 days after inoculation. The hemoglobin content of the matrigel was determined using a Drabkin reagent kit 525 (Sigma). Histological sections on slides were stained with hematoxylin and eosin (HE), and with monoclonal antibodies recognizing endothelial cell marker CD31 and CD34 and the vascular/lymphatic marker VEGF.

### 2.13 Nude mouse xenograft model

Cell aliquots (100 μL) were mixed with matrigel, and the mixture was immediately engrafted into the fourth inguinal mammary fat‐pad of 6‐week old female BALB/c nu/nu mice (n = 8 each group). Tumor sizes were measured every 3 days from the sixth day post‐injection. Six weeks later, all the animals were killed using carbon dioxide asphyxiation and tumor samples were harvested. Informative sections of formalin‐fixed, paraffin‐embedded tumor samples were immunostained with the indicated antibodies. ImmPACT DAB peroxidase substrate (Vector Labs, Burlingame, CA, USA) was used as the chromogen. Hematoxylin was used as counterstain. The sections were photographed using a light microscope OLYMPUS BX‐51. Tumors were also excised for protein extraction for further western blotting analysis.

### 2.14 Immunohistochemical analysis of clinical samples

Surgically resected breast cancer tissues were obtained from 112 patients admitted to the Department of Surgery, Yixing People's Hospital. The patients’ characteristics are detailed in Table S4. The project was approved by the Research Ethics Committee of Nanjing Medical University, and written consent was obtained from each patient enrolled in this study. Two independent investigators performed the immunohistochemical assays of the clinical samples using a semiquantitative scale. The immunohistochemical staining results were scored according to the distribution (0: staining in <5% of tumor cells, 1: staining in 5%–25% of tumor cells, 2: staining in 26%–50% of tumor cells, 3: staining in 51%–75%, 4: staining in >75%) and intensity (0: absent, 1: weak, 2: moderate, 3: strong labeling). Tumors expressing the relevant proteins with scores >2 were designated as ‘high expression’ groups; others were termed as ‘low expression’ groups.

### 2.15 Statistical analysis

Statistical analysis was performed using SPSS 19.0. Results were expressed as the mean ± the standard deviation. Comparisons between two groups were analyzed using a two‐sided Student's *t* test. Correlations between OSX expression and clinicopathological characteristics of the patients with breast cancer were examined using the chi‐squared test. The relationship between OSX and S100A4 expression levels was assessed using Spearman correlation analysis. *P *<* *0.05 was considered statistically significant.

### 2.16 Data availability

All data supporting the findings of this study are available from the corresponding author upon reasonable request.

## 3 RESULTS

### 3.1 OSX promotes breast cancer cell migration and vascular tube formation

**Figure 1:**
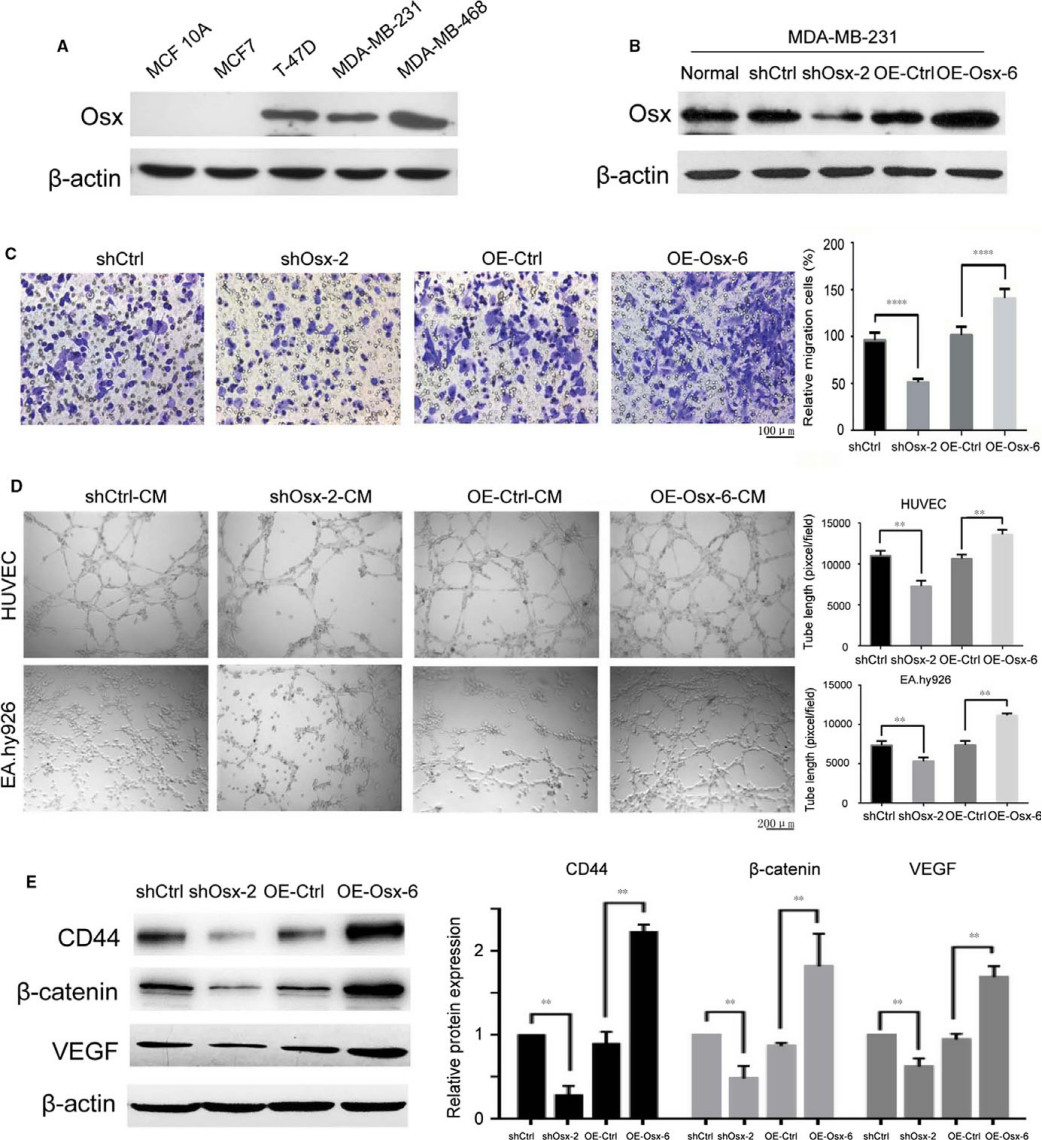
Effects of osterix (OSX) on breast cancer cell migration and endothelial cell tube formation. (A) Differential protein expression of OSX in invasive breast cancer cells T‐47D, MDA‐MB‐231 and MDA‐MB‐468, compared with weakly metastatic breast cancer cells MCF7, and non‐cancerous breast epithelium cells MCF 10A. (B) Stable *OSX* KD and overexpressing MDA‐MB‐231 cells were generated by lentivirus infection. The expression level of OSX was examined by immunoblotting. (C) Cell migration was assessed using a transwell assay. A representative morphological image is shown (left) and the percentage of migrated cells was determined (right). (D) Tube formation by HUVEC and EA.hy926 cells was measured and the results were expressed as the tubule length. Representative morphological images (left) and statistical results (right) are shown. (E) The effects of OSX on the expression levels of CD44, β‐catenin and VEGF were determined by western blotting analysis. The representative figures (left) and the densitometric analysis of the immunoreactive protein bands (right) are presented. Data represent the means ± SD of three independent experiments. CM, conditioned medium. **P <* *0.01. ****P <* *0.0001

Although OSX has been shown to be involved in the regulation of some metastasis‐associated genes, its expression in breast cancer cell lines with different metastatic potencies has not been well surveyed. We initially investigated the expression levels of OSX in a set of breast cancer cell lines with different metastatic features. As shown in Figure 1A, the expression levels of OSX in the more invasive breast cancer cell lines such as T‐47D, MDA‐MB‐231 and MDA‐MB‐468 were much higher than those in the weakly metastatic breast cancer cell line MCF7 and the non‐cancerous breast epithelium cells MCF 10A, suggesting that OSX expression levels correlate positively with the invasion ability of these cell lines.

To determine the role of OSX in the development of breast cancer, we generated MDA‐MB‐231 stable cell lines in which OSX was either knocked down (KD) or overexpressed (OE). OSX expression was obviously decreased in stable knockdown clones 2, 6, and 7 (shOsx‐2, 6, and 7), or increased in overexpressed clones 1, 3, and 6 (OE‐Osx‐1, 3, and 6), compared with their respective control cells (Figure 1B and unpublished data). *OSX* KD inhibited cell migration, while its overexpression promoted cell migration in MDA‐MB‐231 cells, as determined by cell migration assays (Figure 1C and Figure S1A). In addition, we carried out tube formation assay to investigate the effects of OSX on the morphological differentiation of endothelial cells into capillary‐like structures, an important step in the process of angiogenesis.^21^ Treatment of HUVEC and EA.hy926 cells with conditioned medium (CM) harvested from shOsx‐2 cells resulted in broken, shortened and much narrower tube networks, compared with those harvested from shCtrl cells. Conversely, HUVEC and EA.hy926 cells formed a typical blood vessel network when incubated with CM from OE‐Osx‐6 cells (Figure 1D). These results were further confirmed using other *OSX* KD or overexpressing clones (shOsx‐7 and OE‐Osx‐3 cells, Figure S1B).

Dai et al^7^ proposed that expression of VEGF, MMP‐9, β‐catenin, and E‐cadherin was positively regulated by OSX. To determine whether any of these genes, or other genes, could be regulated by OSX in breast cancer cells, we detected the expression levels of certain cell migration‐ and angiogenesis‐related genes in *OSX* KD and overexpressing clones. As predicted, *OSX* KD cells showed markedly reduced CD44 and β‐catenin expression, which are positive regulators of cell migration,^22^, ^23^ and decreased VEGF expression, which is a potent regulator of angiogenesis.^24^ In contrast, overexpression of *OSX* had the opposite effect on the expression levels of CD44, β‐catenin, and VEGF (Figure 1E and Figure S1C).

### 3.2 OSX promotes breast cancer angiogenesis in vivo

**Figure 2:**
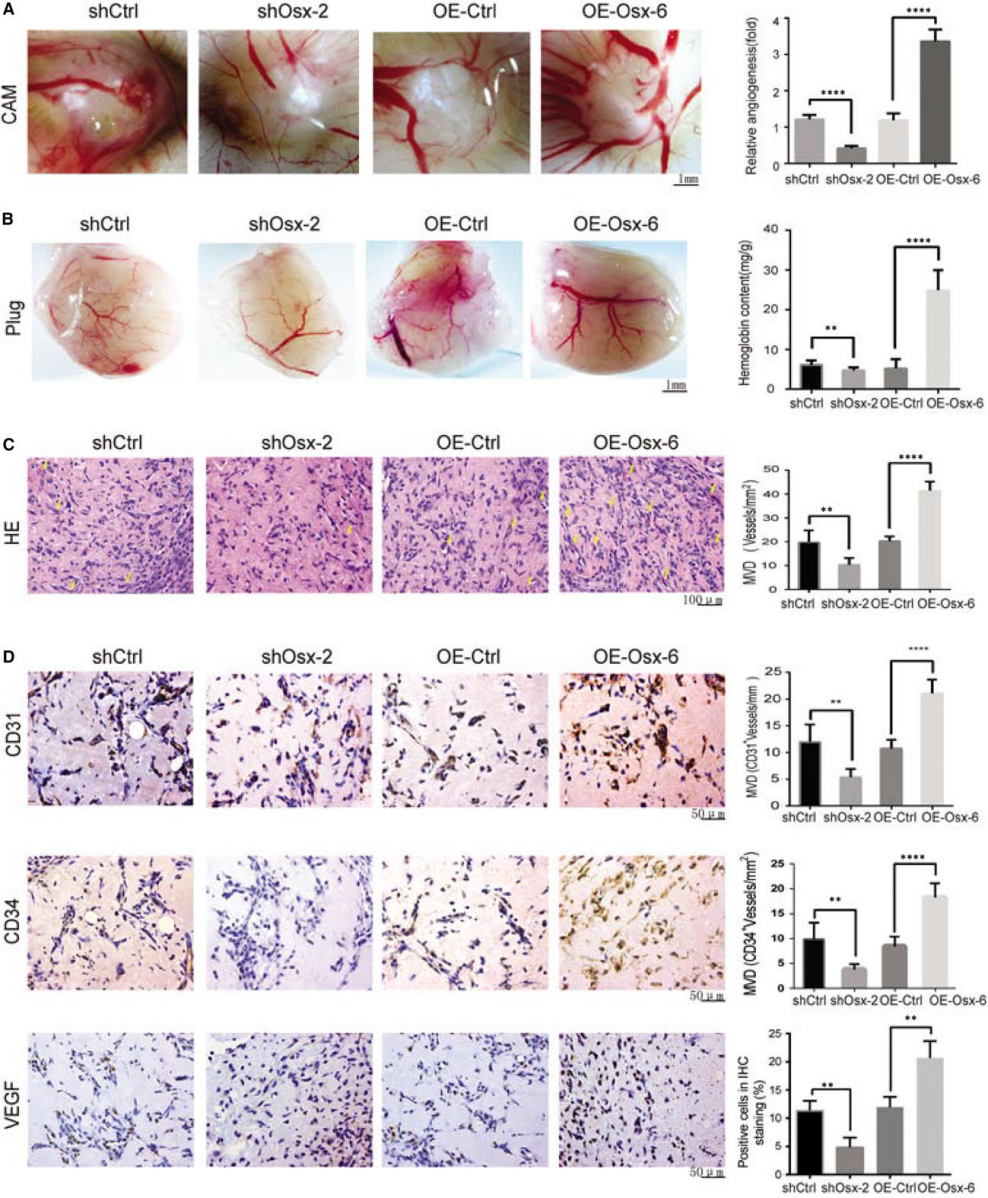
Effects of osterix (OSX) on tumor angiogenesis in vivo. (A) Cells were mixed with matrigel and subsequently implanted onto chicken chorioallantoic membranes (CAMs), as described in Materials and methods. Representative images of angiogenesis on the CAMs are shown. Bar = 1 mm (left). The number of blood vessels was normalized to that of the respective control group, and the results are expressed as the means ± SD (n = 10, right). (B) Cells were mixed with matrigel and injected into right flank of nude mice. Seven days after implantation, gel plugs were collected and photographed (left, bar = 1 mm). Blood vessel formation was quantified by measuring the hemoglobin content using a Drabkin reagent kit 525 (right). (C) Hematoxylin and eosin staining analysis of histological features in plug tissues of nude mice. Arrows point to neovascularization. The right panel shows the quantification of the microvessel density. (D) Immunohistochemical staining analysis of the levels of CD31, CD34 and vascular endothelial growth factor (VEGF) in plug tissues of nude mice. The right panel shows the quantification of the CD31, CD34, and VEGF positive vessels. **P <* *0.01. ****P <* *0.0001

To confirm the in vitro findings, we evaluated the effect of OSX on angiogenesis in the CAM model. As shown in Figure 2A, *OSX* KD reduced the angiogenesis index by 0.3‐fold, while *OSX* overexpression increased the angiogenesis index by 2.8‐fold, compared with their respective control cells. To further verify these results, we investigated the role of OSX in angiogenesis using the matrigel plug assay in nude mice. *OSX* KD significantly reduced blood vessel formation, while *OSX* overexpression led to a marked formation of new blood vessels in the plug, as determined by hemoglobin contents (Figure 2B), microvessel densities (Figure 2C), and the expression levels of vessel positive markers CD31, CD34, and VEGF (Figure 2D).

### 3.3 OSX promotes cell migration and angiogenesis through upregulating S100A4 levels

**Figure 3:**
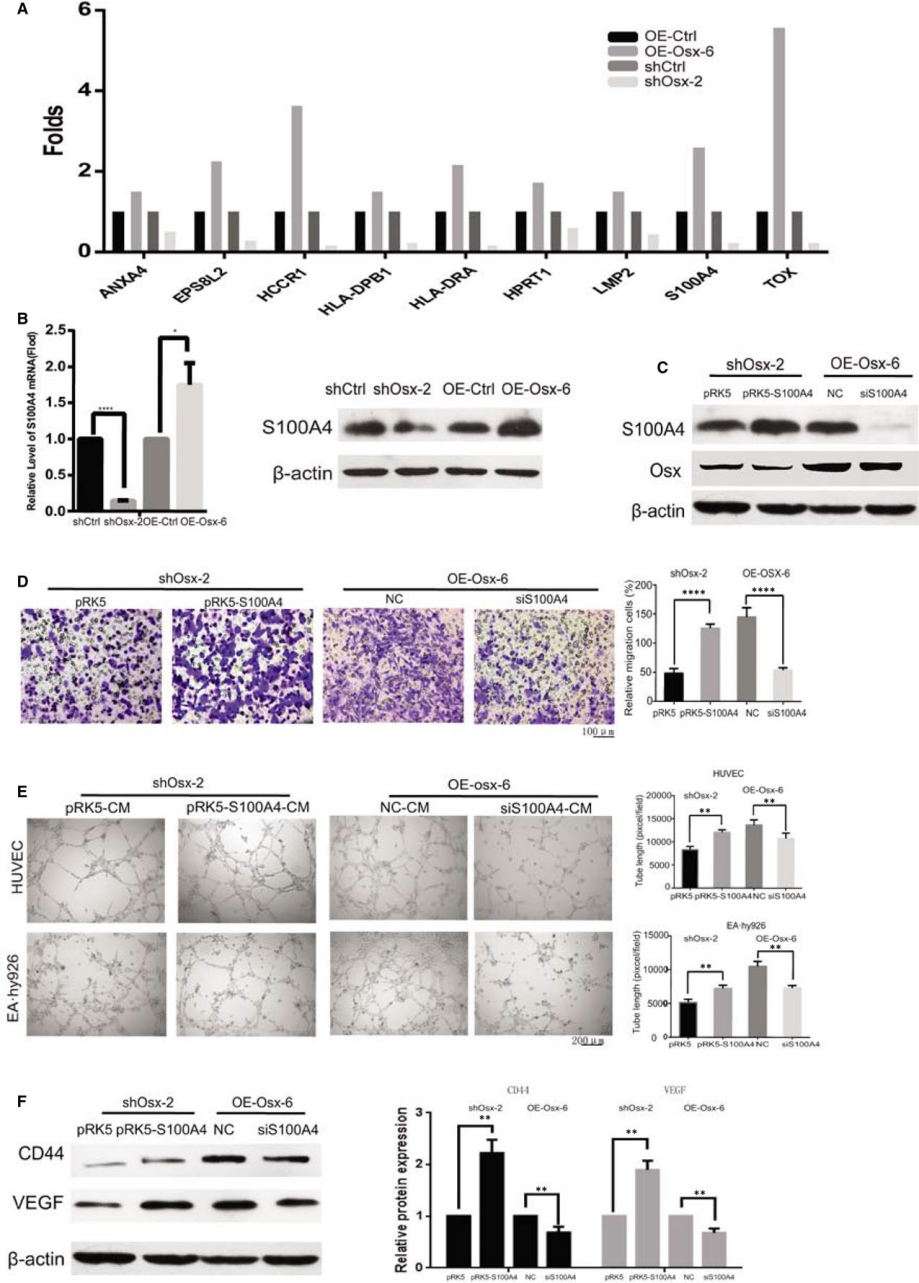
*S100A4* is the target gene in osterix (OSX)‐induced cell migration and angiogenesis. (A) Mass spectrometric analysis of differentially expressed proteins in shOsx‐2, OE‐Osx‐6, and their respective control cells. A total of nine cancer‐related genes are shown. (B) The mRNA and protein expression levels of S100A4 in shOsx‐2, OE‐Osx‐6, and their respective control cells were determined by qRT‐PCR (left) and western blotting (right). (C) The expression levels of OSX and S100A4 were determined by western blotting analysis in shOsx cells transfected with a construct expressing S100A4 and in OE‐Osx cells treated with an S100A4‐siRNA. (D) Cell migration was assessed using a transwell assay. A representative morphological image is shown (left) and the percentage of migrated cells was determined (right). (E) Tube formation by HUVEC and EA.hy926 cells was measured and the results were expressed as the tubule length. Representative morphological images (left) and statistical results (right) are shown. (F) The expression levels of CD44 and vascular endothelial growth factor (VEGF) were determined by western blotting analysis. The representative figures (left) and the densitometric analysis of the bands (right) are presented. Data represent the means ± SD of three independent experiments. CM, conditioned medium. **P <* *0.01. ****P <* *0.0001

To further probe the mechanism of how OSX contributes to breast cancer cell migration and tumor angiogenesis, a mass spectrometry‐based proteomics method was employed. A total of 19 differentially expressed proteins were identified in shOsx and OE‐Osx cells, compared with their levels in their respective controls. Twelve of them were downregulated in shOsx cells and upregulated in OE‐Osx cells, and the other seven proteins were upregulated in shOsx cells and downregulated in OE‐Osx cells (Table 1 and Table S5). Among them, nine proteins, including ANXA4,^25^ EPS8L2,^26^ HCCR1,^27^ HLA‐DPB1,^28^ HLA‐DRA1,^29^ HPRT1,^30^ LMP2,^31^ S100A4^32^ and TOX,^33^ have been reported to be involved in cancer progression as shown in Figure 3A. Growing evidence indicates that elevated S100A4 protein levels are associated with the progression and angiogenesis of several malignant tumors, including breast cancer,^17^ non‐small cell lung cancer,^18^ prostate cancer,^19^ and colon cancer.^34^ Therefore, we hypothesized that OSX might exert its effects on cell migration and angiogenesis by regulating S100A4 expression in breast cancer. To test this hypothesis, the changes in S100A4 expression were further assessed by qRT‐PCR and western blotting. *OSX* KD significantly reduced both the mRNA and protein levels of S100A4, whereas *OSX* overexpression had the opposite effect (Figure 3B and Figure S2).

**Table 1:** The list of differentially abundant proteins in shOSX and OE‐OSX cells, compared with their levels in their respective controls

Protein name	shOsx‐2/shCtrl		OE‐Osx‐6/OE‐Ctrl	
	Fold change	*P*‐value	Fold change	*P*‐value
ANXA4	0.523	0.011	1.501	0.003
ATP5G3	2.118	<0.001	0.552	0.002
EPS8L2	0.307	0.037	2.297	0.012
HCCR	0.168	0.011	3.649	0.005
HLA‐DRA	0.155	0.001	2.168	0.005
HPRT1	0.592	0.011	1.719	0.010
HLA‐DPB1	0.248	0.007	1.529	0.042
HLA‐DRB1	0.105	0.002	4.925	0.001
HMOX1	3.342	<0.001	0.357	0.048
KRT7	0.626	0.001	1.119	0.001
LMP2	0.671	0.010	1.507	0.002
NACC1	1.584	0.037	0.659	0.026
PTTG1IP	2.287	0.024	0.576	0.023
S100A4	0.220	0.003	2.615	0.002
OSX	0.267	<0.001	17.446	<0.001
SPANXB1	4.317	0.039	0.549	0.033
SRGN	4.977	0.004	0.403	0.004
TARSL2	0.041	<0.001	3.541	<0.001
TOX	0.249	0.012	5.567	0.008
ZNF891	1.742	0.003	0.632	0.005

We then evaluated the involvement of S100A4 in OSX‐induced cell migration and angiogenesis of breast cancer. Three siRNA oligonucleotides targeting different sites in the mRNA of S100A4 were tested and S100A4‐siRNA‐1 was selected for further experimental because of its highly efficient knockdown of S100A4 expression (Figure S3). S100A4 expression was restored by transfection of a construct expressing S100A4 in shOsx cells, while it was depleted using S100A4‐siRNA‐1 in OE‐Osx cells. There was no change in the expression of endogenous OSX (Figure 3C). Restored expression of S100A4 significantly rescued OSX‐shRNA‐suppressed cell migration and capillary‐like tube formation. In contrast, S100A4 KD impaired OSX‐induced cell migration and capillary‐like tube formation, as determined by transwell migration and tube formation assays (Figure 3D,E). These data strongly suggested that OSX‐induced cell migration and capillary‐like tube formation was partially mediated by S100A4.

Interestingly, the decrease in CD44 and VEGF expression was also abolished after restoring the expression of S100A4 in shOsx cells, and increased CD44 and VEGF levels were downregulated in S100A4 depleted OE‐Osx cells (Figure 3F and Figure S4). There was no change in the expression of β‐catenin after restoring S100A4 in shOsx cells or in S100A4‐depleted OE‐Osx cells (data not shown). These data suggested that S100A4 induces cell migration and angiogenesis partially via modulation of CD44 and VEGF in breast cancer cells.

### 3.4 OSX induces migration and angiogenesis‐related genes expression in vivo

**Figure 4:**
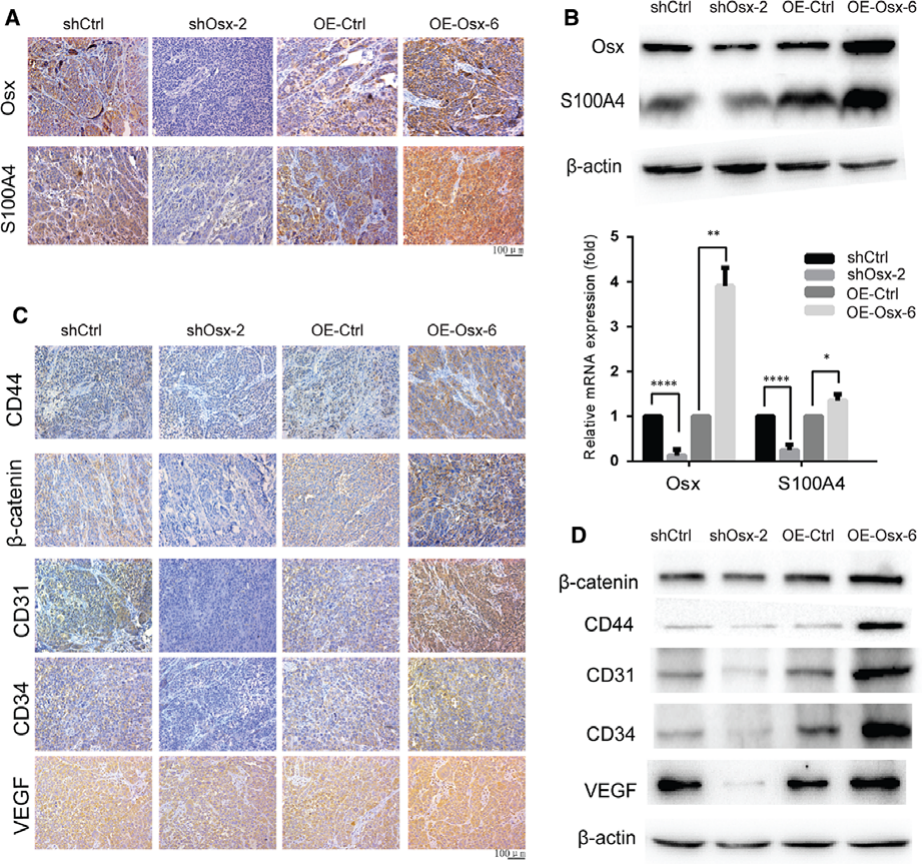
Effects of osterix (OSX) on the expression of migration and angiogenesis‐related genes in vivo. (A) Cells were mixed with matrigel and engrafted into the fourth inguinal mammary fat‐pads of nude mice. Six weeks later, the tumor samples were harvested. Immunohistochemical analysis was used to detect the expression levels of OSX and S100A4 in nude mice tumors. (B) Relative mRNA and proteins expression levels of OSX and S100A4 were determined by qRT‐PCR and western blotting analysis, respectively, in nude mice tumors. (C) Immunohistochemical staining analysis was used to detect the expression levels of CD44, β‐catenin, CD31, CD34 and vascular endothelial growth factor (VEGF) in nude mice tumors. (D) The protein expression levels of CD44, β‐catenin, CD31, CD34 and VEGF were analyzed by western blotting analysis in protein samples from nude mice tumors. *P <* *0.05. **P <* *0.01. ****P <* *0.0001

To further validate the effects of S100A4 on OSX‐induced cell migration and angiogenesis in vivo, *OSX* KD or overexpressing cells were injected into the fourth mammary fat‐pad of BALB/c‐nu/nu mice. Tumor volumes were measured every 3 days when they were palpable. Tumors from the shOsx group were significantly smaller than those from control group. However, there was no significant difference in tumor volume between the OE‐Osx and control group (data not shown). As expected, the expression of S100A4 was markedly reduced in tumors from the shOsx group, and significantly increased in tumors from the OE‐Osx group (Figure 4A,B). In addition, immunohistochemical staining and western blotting analysis showed that the expression levels of migration‐related proteins CD44, β‐catenin, the endothelial markers CD31 and CD34, and the vascular/lymphatic marker VEGF were all decreased in tumors from the shOsx group and increased in tumors from the OE‐Osx group (Figure 4C,D). Collectively, these data showed that knockdown of OSX inhibited migration and angiogenesis by downregulating S100A4, CD44, β‐catenin, CD31, CD34, and VEGF levels in vivo, whereas overexpression of OSX had the opposite effect.

### 3.5 OSX expression is positively correlated with S100A4 levels in breast cancer tissues

**Figure 5:**
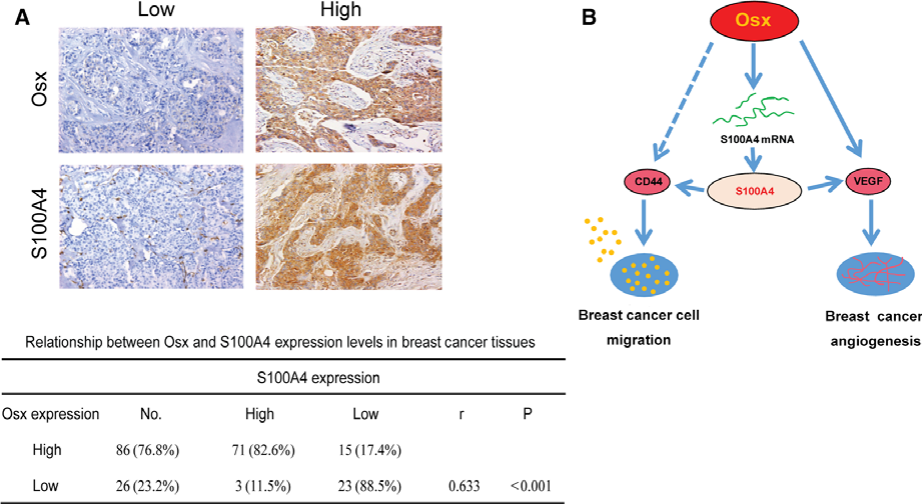
Correlations between osterix (OSX) and S100A4 expression levels in breast cancer tissues. (A) Representative immunohistochemical peroxidase staining for OSX and S100A4 in breast cancer tissues (upper) and statistic data of OSX and S100A4 expression levels (lower). (B) Schematic diagram representing the role of OSX in cell migration and tumor angiogenesis in breast cancer

Using immunohistochemical staining, we examined whether OSX expression is correlated with S100A4 expression in breast cancer samples. As shown in Figure 5A, high expression of OSX was detected in 86 cases, among which 71 cases exhibited high expression of S100A4 (82.6%). Meanwhile, there were 26 cases with low expression of OSX, among which 23 cases showed low expression of S100A4 (88.5%). By contrast, there was no statistically significant correlation between OSX expression and the patients’ age, tumor size, or histological sub‐type (ER, PR, or HER2 status) (Table S6). These results strongly indicated that the expression levels of OSX are significantly and positively correlated with those of S100A4 in breast cancer tissues.

## 4 DISCUSSION

To date, there have been few reports regarding OSX and tumorigenesis. The present study provided in vitro and in vivo evidence to support the view that OSX plays important roles in cell migration and tumor angiogenesis in breast cancer. We demonstrated that OSX facilitates cell migration and angiogenesis in breast cancer, such as promotion breast cancer cell migration using a transwell assay, induction of tube‐like structure formation using a tube formation assay, and formation of new blood vessels in a CAM assay. Moreover, the in vivo matrigel plug model also supported our in vitro observations that OSX promotes breast cancer angiogenesis. Some studies have also identified the cancer‐promoting effects of OSX in different types of cancers, including human osteosarcomas and prostate cancer.^5^, ^6^ Nevertheless, in a study based on a mouse osteosarcoma model, Osx was down‐regulated and its expression was negatively associated with metastatic potency.^35^ This discrepancy may be caused by the context dependence of the specific cell lines used in each study.

A mass spectrometry‐based analysis identified 19 differentially expressed proteins in shOsx and OE‐Osx cells, compared with their respective controls. We decided to focus on S100A4 because it is a mediator of tumor cell migration and angiogenesis.^12^, ^13^, ^14^ The results of in vivo xenograft mouse experiments also supported our in vitro observation that OSX regulates S100A4. Importantly, restored expression of S100A4 significantly rescued OSX‐shRNA‐suppressed cell migration and capillary‐like tube formation, while S100A4 KD inhibited OSX‐induced cell migration and capillary‐like tube formation. These data strongly suggested that OSX‐induced cell migration and capillary‐like tube formation were partially mediated by S100A4. Moreover, OSX expression was significantly positively correlated with the level of S100A4 in breast cancer tissues. An increase in S100A4 protein expression has been correlated with a worse prognosis for patients with different types of cancer including breast, colon, gastric, lung, hepatocellular, and pancreatic cancer,^34^, ^36^, ^37^, ^38^, ^39^ which suggested that OSX could be used as a target gene to improve cancer prognosis.

As shown above, S100A4 was differentially expressed at the mRNA and protein levels between *OSX* KD and *OSX* overexpressing cells, compared with their respective control cells. The differential expression of S100A4 might occur by three possible mechanisms: differential mRNA transcription, mRNA stability, or protein stability of S100A4. OSX belongs to the specificity protein (SP) family that is presumed to function by binding directly to DNA promoter elements via an SP1‐like DNA‐binding domain.^4^ The human *S100A4* gene contains four exons, two of which are non‐coding at the 5′ UTR position^14^; the transcription of the *S100A4* gene is controlled by both positive and negative regulatory elements located within the first intron, which is bound by several transcript factors.^40^, ^41^ Bioinformatic analyses indicated that the core region of the *S100A4* promoter contains five potential OSX‐binding sites (data not shown). We speculated that OSX probably regulates the transcription of *S100A4* by binding to its promoter. Nevertheless, further investigation is required to uncover the detailed mechanisms by which OSX regulates *S100A4* expression.

A previous study revealed that OSX was associated with the expression of a number of metastasis‐associated genes such as *VEGF*,* MMP‐9*, β*‐catenin*, and *E‐cadherin*.^7^, ^42^ VEGF is one of the most potent endothelial cell mitogens and plays a crucial role in tumor growth, angiogenesis and metastasis.^43^, ^44^, ^45^ It has been reported that VEGF is the direct target gene of OSX in osteoblasts.^42^ In our study, we found that OSX positively regulated VEGF expression in breast cancer cells. More interestingly, the decrease in VEGF expression was abolished after restoring the expression of S100A4 in shOsx cells, and increased VEGF was downregulated in S100A4 depleted OE‐Osx cells. It has been reported that S100A4 alters the neovascularization ability in tumors by regulating VEGF.^46^ A significant relationship between S100A4 and VEGF expression was also demonstrated in clear renal cell carcinoma, gastric carcinoma and pancreatic cancer.^47^, ^48^, ^49^ Therefore, OSX induces angiogenesis at least in part through the S100A4‐VEGF pathway (Figure 5B). In our study, we also found OSX positively regulated CD44 expression, and the decrease in CD44 expression was abolished after restoring the expression of S100A4 in shOsx cells while increased CD44 expression was downregulated in S100A4 depleted OE‐Osx cells. CD44 participates in many cellular processes, including the regulation of cell survival, migration, and adhesion through the binding of its major ligand, hyaluronic acid.^50^ Aberrant overexpression of CD44 correlates with the metastatic potential of several malignant tumors, such as prostate cancer, breast tumors and chondrosarcoma^.^ 22, 51, 52 S100A4 induced the re‐distribution of CD44 and enhanced the cell surface expression of CD44, thereby inhibiting cell‐cell and cell‐matrix adhesion in B16 murine melanoma cells.^53^ In addition, transfection with S100A4 siRNA significantly reduced the expression of CD44 in osteosarcoma cells.^54^ These data suggested that OSX might induce migration partly through the S100A4‐CD44 pathway (Figure 5B).

In conclusion, this study revealed that OSX could potentiate breast cancer cell migration and tumor angiogenesis by up‐regulating S100A4 expression in vitro and in vivo. Augmented CD44 and VEGF in breast cancer cells are associated with OSX‐mediated cell migration and angiogenesis. Overall, our study suggested that OSX participates in breast cancer malignancy and might serve as a potential target for breast cancer therapy.

## CONFLICT OF INTEREST

The authors confirm that there are no conflicts of interest.

## AUTHOR CONTRIBUTION

SQ and JW performed the major proportion of the experiments; QB, BY and RD performed parts of the research; XC collected the clinical samples; SQ, LL, HY and YJ analyzed the data, tested the statistics, and coordinated the figures; YJ and CM were responsible for study design, data analysis and writing the manuscript.
