# Supplementary figures and images for: Osterix promotes the migration and angiogenesis of breast cancer by upregulation of S100A4 expression
Source: J Cell Mol Med. 2018 Nov 18;23(2):1116–27. doi: 10.1111/jcmm.14012 (PMC6349213; doi:10.1111/jcmm.14012)

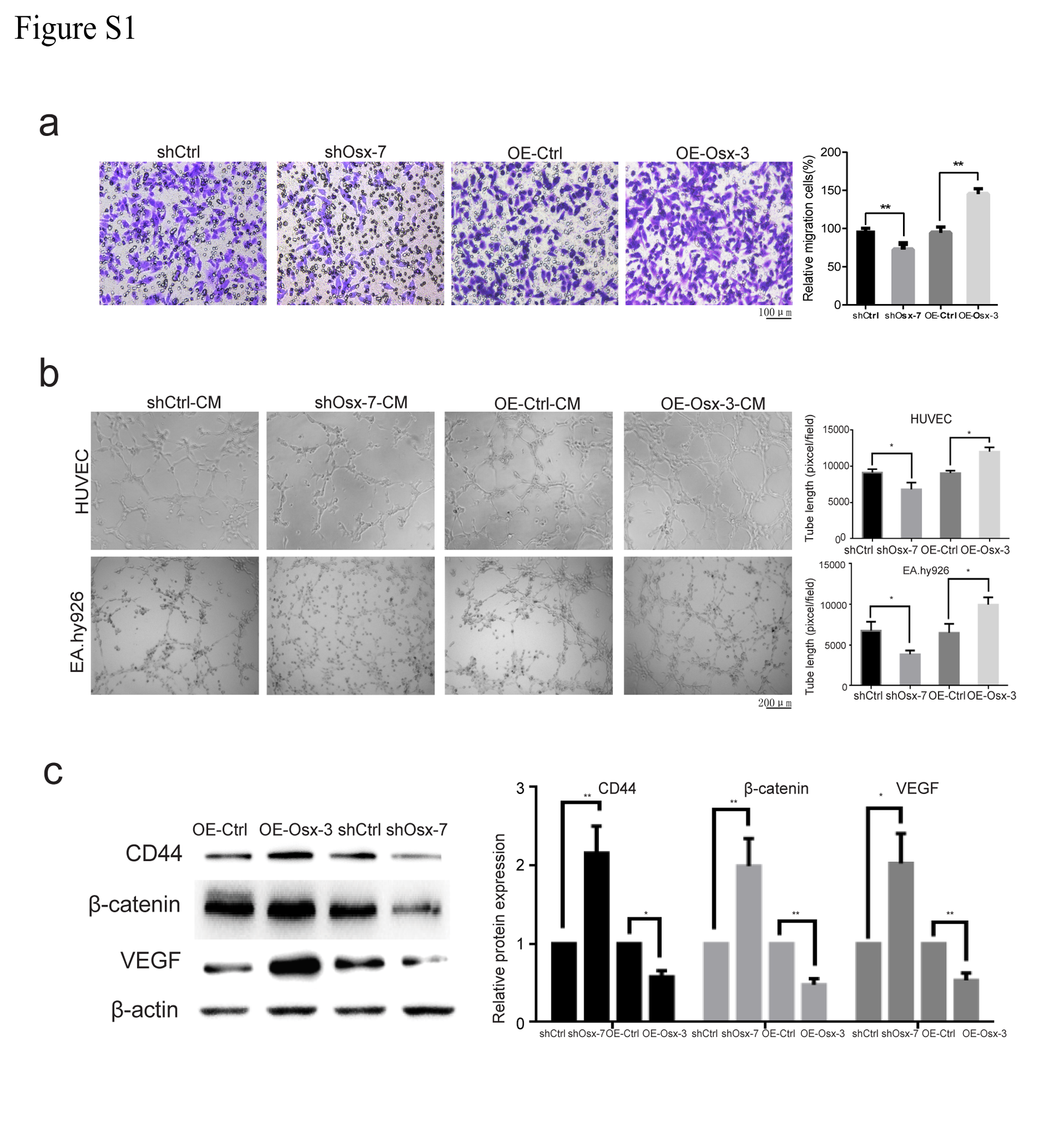

Supplement: Supplementary file 1 [file JCMM-23-1116-s001.tif]

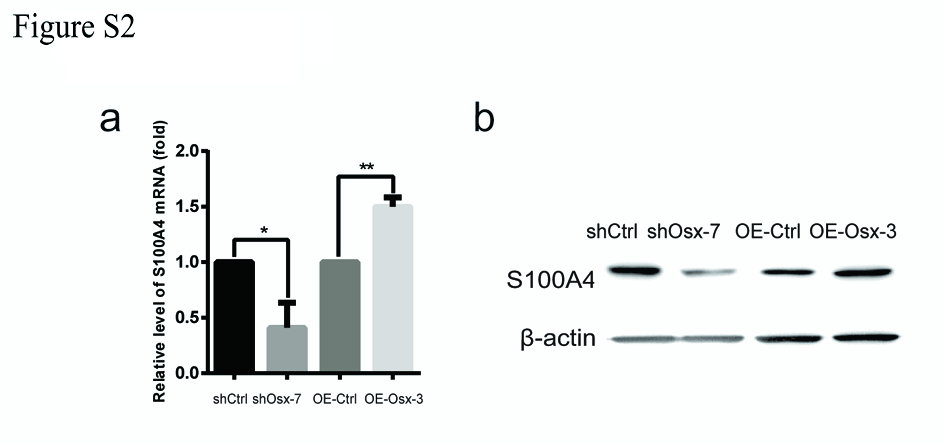

Supplement: Supplementary file 2 [file JCMM-23-1116-s002.tif]

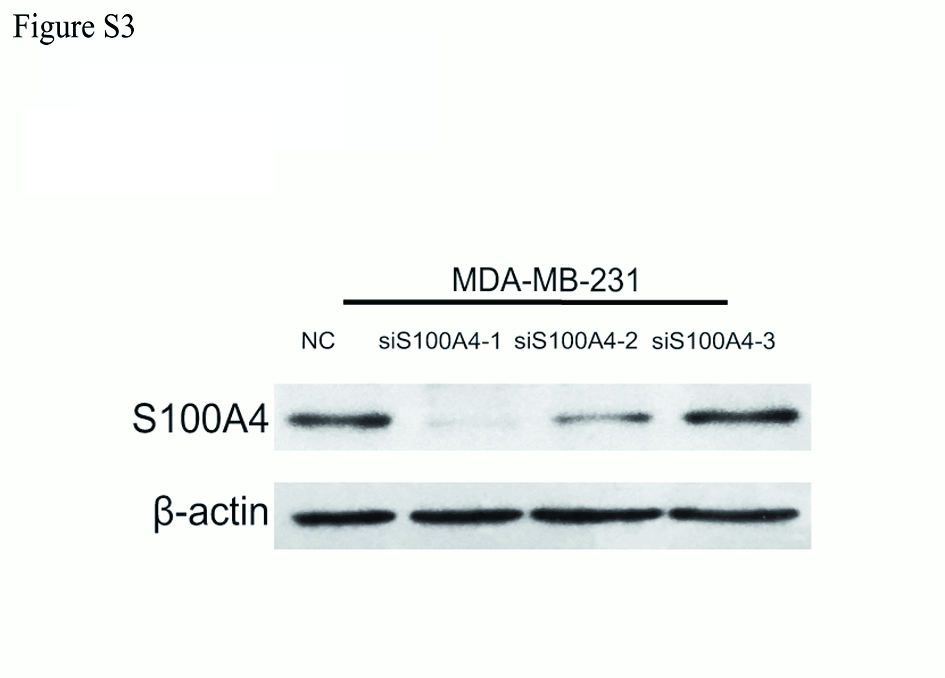

Supplement: Supplementary file 3 [file JCMM-23-1116-s003.tif]

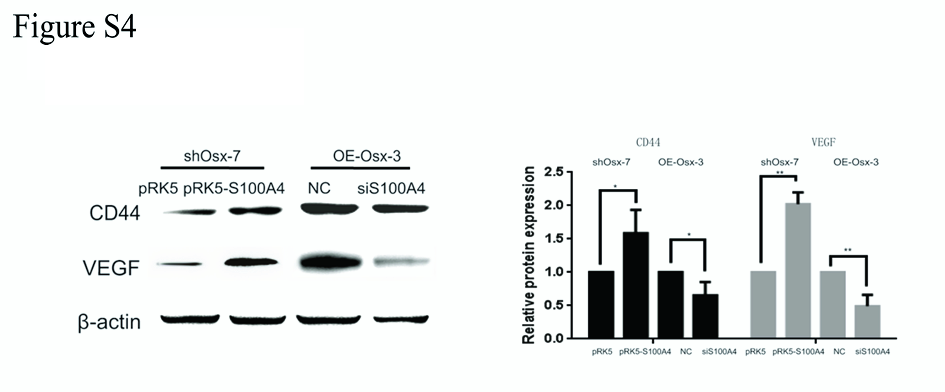

Supplement: Supplementary file 4 [file JCMM-23-1116-s004.tif]
