# Supplementary material for: Osterix promotes the migration and angiogenesis of breast cancer by upregulation of S100A4 expression
Source: J Cell Mol Med. 2018 Nov 18;23(2):1116–27. doi: 10.1111/jcmm.14012 (PMC6349213; doi:10.1111/jcmm.14012)
Supplement: Supplementary file 5 [file JCMM-23-1116-s005.docx]

**Table S1. Primers used for plasmid constructs**

| Gene names | Forward/Reverse | Sequences 5'-3' |
| --- | --- | --- |
| *OSX* cDNA | Forward | CGGGATCCATGGCGTCCTCCCTGCTTGA |
|  | Reverse | CCCTCGAGTCAGATCTCCAGCAAGTTGC |
| *S100A4* cDNA | Forward | CCATCGATATGGCGTGCCCTCTGGAG |
|  | Reverse | CCCAAGCTTTCATTTCTTCCTGGGCTGC |
