# Supplementary material for: Osterix promotes the migration and angiogenesis of breast cancer by upregulation of S100A4 expression
Source: J Cell Mol Med. 2018 Nov 18;23(2):1116–27. doi: 10.1111/jcmm.14012 (PMC6349213; doi:10.1111/jcmm.14012)
Supplement: Supplementary file 6 [file JCMM-23-1116-s006.docx]

**Table S2.** **Sequences used for knockdown of target genes**

| Gene names | Sequence number | Sequences 5'-3' |
| --- | --- | --- |
| *OSX* | #1 | ACAAGCACTAATGGGCTCCTTCTCGAGAAGGAGCCCATTAGTGCTTGT |
|  | #2 | CCCAAGATGTCTATAAACCCACTCGAGTGGGTTTATAGACATCTTGGG |
|  | #3 | CCTCAGGCTATGCTAATGATTCTCGAGAATCATTAGCATAGCCTGAGG |
