# Supplementary material for: Osterix promotes the migration and angiogenesis of breast cancer by upregulation of S100A4 expression
Source: J Cell Mol Med. 2018 Nov 18;23(2):1116–27. doi: 10.1111/jcmm.14012 (PMC6349213; doi:10.1111/jcmm.14012)
Supplement: Supplementary file 7 [file JCMM-23-1116-s007.docx]

**Table S3. Primers used for qRT-PCR assay**

| Gene names | Forward/Reverse | Sequences 5'-3' |
| --- | --- | --- |
|  |  |  |
| *S100A4* | Forward | CTGACTGCTGTCATGGCGT |
|  | Reverse | ACCACATCAGAGGAGTTTTCAT |
| *OSX* | Forward | CTCCTTTCACCTGCAGGCAG |
|  | Reverse | CAGACAGTCAGAAGAGCTGT |
| *β-actin* | Forward | AGATGTGGATCAGCAAGCAG |
|  | Reverse | GCGCAAGTTAGGTTTTGTCA |
