# Supplementary material for: Osterix promotes the migration and angiogenesis of breast cancer by upregulation of S100A4 expression
Source: J Cell Mol Med. 2018 Nov 18;23(2):1116–27. doi: 10.1111/jcmm.14012 (PMC6349213; doi:10.1111/jcmm.14012)
Supplement: Supplementary file 10 [file JCMM-23-1116-s010.docx]

**Table S6. Correlation between OSX expression and clinicopathological features in breast cancer (n = 112)**

| Variable | Cases | OSX expression | | *P* Value^a^ |
| --- | --- | --- | --- | --- |
|  |  | Low | High |  |
| **Age** |  |  |  |  |
| ≤ 50 yr | 48 | 10 | 38 | 0.576 |
| ＞50 yr | 64 | 16 | 48 |  |
| **Tumor size** |  |  |  |  |
| ≤ 2 cm | 61 | 15 | 46 | 0.750 |
| ＞2 cm | 51 | 11 | 40 |  |
| **HER2 status** |  |  |  |  |
| Negative | 34 | 10 | 24 | 0.324 |
| Positive | 78 | 16 | 62 |  |
| **ER status** |  |  |  |  |
| Negative | 34 | 4 | 30 | 0.055 |
| Positive | 78 | 22 | 56 |  |
| **PR status** |  |  |  |  |
| Negative | 47 | 7 | 40 | 0.070 |
| Positive | 65 | 19 | 46 |  |

^a^Chi-squared detection.
